# Renal cell carcinoma with tumor thrombus growing against the direction of venous return: an indicator of complicated surgery and poor prognosis

**DOI:** 10.1186/s12893-021-01448-0

**Published:** 2021-12-28

**Authors:** Zhuo Liu, Yuxuan Li, Xun Zhao, Liyuan Ge, Guodong Zhu, Peng Hong, Shiying Tang, Shudong Zhang, Xiaojun Tian, Shumin Wang, Cheng Liu, Hongxian Zhang, Lulin Ma

**Affiliations:** 1 Department of Urology, Peking University Third Hospital, 49 North Garden Road, Haidian, Beijing, 100191 People’s Republic of China; 2 Department of Ultrasound, Peking University Third Hospital, Haidian District, Beijing, 100191 People’s Republic of China

**Keywords:** Renal cell carcinoma, Tumor thrombus, Surgery, Prognosis, Growing against the direction of venous return

## Abstract

**Purpose:**

To explore the effect of tumor thrombus growing against the direction of venous return (GADVR) tumor thrombus on the choice of surgical approach, the impact on the complexity of the surgery and the prognosis.

**Methods:**

We retrospectively analyzed the clinicopathological data of 213 patients, who underwent surgery in a single center of Peking University Third Hospital between January 2016 and June 2020. For right renal cell carcinoma (RCC) and venous tumor thrombus (VTT), imaging revealed a filling defect in the left renal vein, which was significantly enhanced in enhanced imaging, suggesting that the tumor thrombus grew against the direction of venous return into the left renal vein. For left RCC and VTT, at least one of the left renal vein branches has tumor thrombus. The branches include the left adrenal vein, the left gonadal vein (testicular vein or ovarian vein), and the left ascending lumbar vein. The patients were divided into two groups according to whether they were GADVR tumor thrombus, and we compare the clinicopathological characteristics of GADVR tumor thrombus and non-GADVR tumor thrombus. Univariate and multivariate Cox regression analyses were performed to explore the risk factors that affect the prognosis of patients with RCC and VTT. Kaplan–Meier plots were conducted to evaluate the effect of GADVR on progression-free survival (PFS).

**Results:**

Compared with non-GADVR tumor thrombus, patients with GADVR tumor thrombus had a higher proportion of open surgery (76.2% vs. 52.1%, P = 0.035), a higher proportion of tumor thrombus adhering to the inferior vena cava (IVC) vessel wall (81% vs. 45.8%, P = 0.002), a higher proportion of segmental resection of the IVC vessel wall (61.9% vs. 15.6%, P < 0.001); higher preoperative serum creatinine value (110.0 μmol/L vs. 91.0 μmol/L, P = 0.015), a higher proportion of tumor thrombus combined with bland thrombus (non-tumor thrombus) (57.1% vs. 19.8%, P < 0.001). In terms of surgical complexity, patients with GADVR tumor thrombus had a longer median operation time (379 min vs. 308 min, P = 0.038), more median surgical blood loss (1400 mL vs. 600 mL, P = 0.018), and more postoperative complications (52.4% vs. 30.7%, P = 0.045). Multivariate Cox regression analysis showed that GADVR tumor thrombus, symptoms, postoperative serum creatinine, distant metastasis, sarcomatoid feature, pathological type, lymph node dissection were independent risk factors for PFS. Patients with GADVR tumor thrombus’s median survival time was 14.0 months, while patients with non-GADVR tumor thrombus were 32.0 months (P = 0.016). GADVR tumor thrombus is an independent risk factor for PFS in patients with RCC and VTT.

**Conclusion:**

GADVR tumor thrombus is a characteristic feature of tumor thrombus, with an incidence of 9.9%. It has a higher proportion of open surgery and higher surgical complexity, which is an independent risk factor for PFS.

## Introduction

Renal cell carcinoma (RCC) is a common malignant tumor of the urinary system. The incidence of RCC in China in 2015 was 3.35 per 100,000 [1], and in the past two decades, the incidence rate has increased by about 2% each year [2]. One of the essential clinical features of RCC is that it often invades the renal vein and inferior vena cava (IVC) and forms venous tumor thrombus, which accounts for about 4–10% of RCC patients [3]. The overall prognosis of patients with RCC and VTT is poor, and the 5-year tumor-specific survival rate is only 25–53% [4, 5]. Radical nephrectomy with thrombectomy is the only possible radical treatment for non-metastatic RCC with IVC tumor thrombus, but the 5-year survival rate is still 40–65% [6, 7]. Clarifying the growth characteristics of tumor thrombus has essential clinical value for diagnosing and treating this type of disease. According to the traditional view, the growth process of tumor thrombus in the IVC is as follows: primary renal tumor cells proliferate continuously, break through the capillary endothelial and enter the blood vessels, through the direction of venous return, grow along the affected renal vein and enter the IVC [8–10]. Tumor thrombus in a few patients may enter the right atrium through the IVC [11, 12]. However, in the clinical practice, we found a particular type of patients whose tumor thrombus grew against the direction of venous return. We defined it as tumor thrombus growing against the direction of venous return (GADVR) tumor thrombus. The right renal vein usually has no extrarenal branches. The left renal vein receives the left adrenal vein, left testis, or ovarian vein. In some patients, the left renal vein is connected to the left ascending lumbar vein [13]. For the right RCC and VTT, the GADVR tumor thrombus is manifested as the left renal vein tumor thrombus. For left RCC and VTT, the GADVR tumor thrombus showed left adrenal vein, left gonadal vein (testicular or ovarian vein), and left lumbar ascending vein tumor thrombus. We found that this GADVR tumor thrombus is rare in clinical practice, but its operation is more complex, and the prognosis is worse. For further validation, we retrospectively analyzed the clinicopathological data of 213 patients admitted to the single center from January 2016 to June 2020. They were divided into two groups according to whether they were GADVR tumor thrombus.

## Materials and methods

**Fig. 1:**
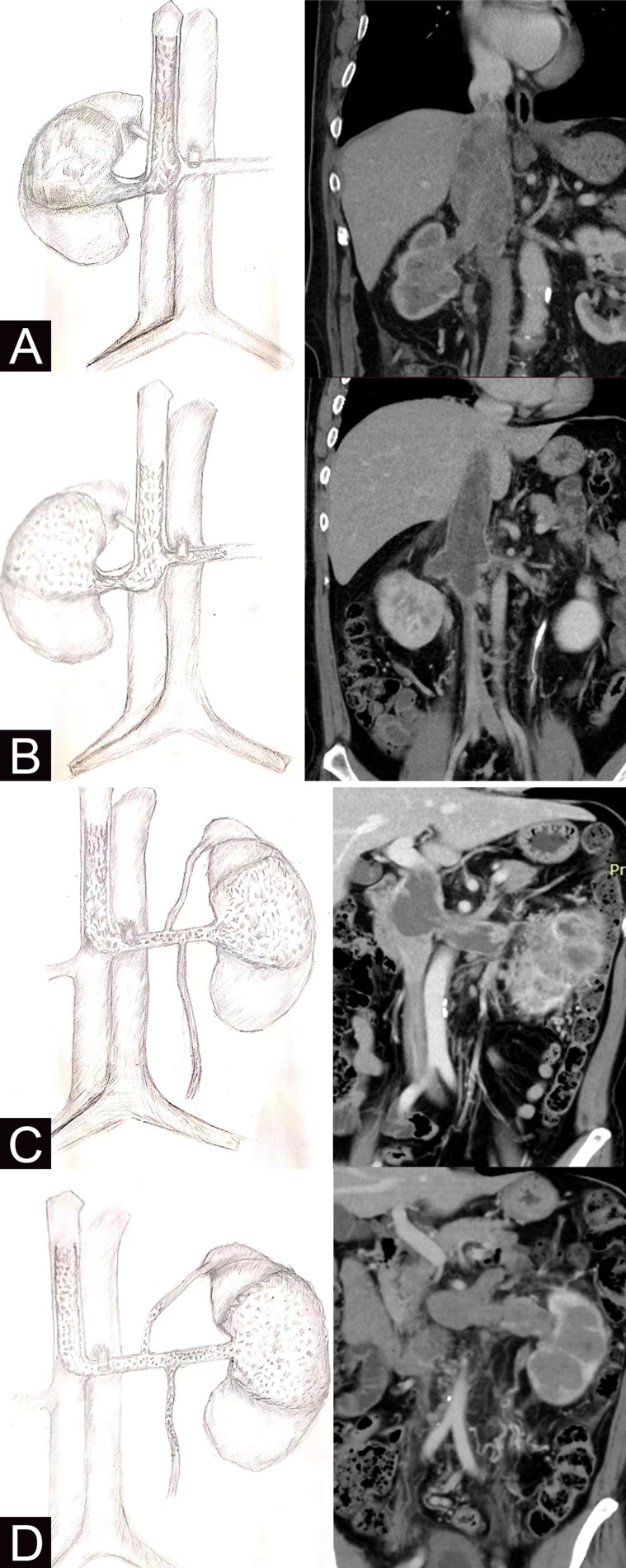
Morphological diagram of GADVR tumor thrombus and non-GADVR tumor thrombus and enhanced CT examination of the urinary system in typical patients (coronal position): The left image was diagram and the right image was enhanced CT examination of the urinary system (coronal position). **A** Right RCC with IVC tumor thrombus without GADVR tumor thrombus: The tumor occurs from the right kidney, forming tumor thrombus in the right renal vein, and grows into the IVC with the direction of venous return. **B** Right RCC with IVC tumor thrombus accompanied by GADVR tumor thrombus: The tumor occurs from the right kidney and forms tumor thrombus in the right renal vein. In addition to part of the tumor grows in the IVC with the direction of venous return, another part grows in the left renal vein against the direction of venous return or in the right gonadal vein against the direction of venous return. **C** Left RCC with IVC tumor thrombus without GADVR tumor thrombus: The tumor occurs from the left kidney, forming tumor thrombus in the left renal vein, and grows into the IVC with the direction of venous return. **D** Left RCC with IVC tumor thrombus accompanied by GADVR tumor thrombus: The tumor occurs from the left kidney and forms tumor thrombus in the left renal vein. In addition to part of the tumor grows into the IVC with the direction of venous return, another part of the tumor grows into the left renal vein branches such as the left adrenal vein, gonadal vein, and ascending lumbar vein, against the direction of venous return

Inclusion criteria were as follows: (a) preoperative imaging examination, urinary enhanced CT, and/or IVC enhanced MRI showed RCC with renal vein or IVC tumor thrombus. (b) For right RCC and VTT, imaging revealed a filling defect in the left renal vein or right gonadal vein, which was significantly enhanced in enhanced imaging, suggesting that the tumor thrombus grew against the venous return into the left renal vein or the right gonadal vein. For left RCC and VTT, at least one of the left renal vein branches has tumor thrombus. The branches include the left adrenal vein, the left gonadal vein (testicular vein or ovarian vein), and the left ascending lumbar vein. In addition to imaging findings indicating GADVR tumor thrombus’ presence, it is necessary to explore its presence during surgery and confirm the tumor thrombus by postoperative pathological diagnosis (see Fig. 1). The exclusion criteria were as follows: (a) GADVR type tumor thrombus was considered preoperatively and confirmed by postoperative pathological diagnosis as bland thrombus (non-neoplastic). (b) Patients without surgery. (c) Postoperative pathological type was non-renal cell carcinoma. Patients’ clinical manifestations were divided into local symptoms such as low back pain, hematuria, abdominal mass, and systemic symptoms such as fever and weight loss. The surgical methods were divided into laparoscopic minimally invasive approach and open approach. Tumor thrombus classification is divided into five categories according to the position of the proximal end of the tumor thrombus using Mayo classification [14]. Inferior vena cava MRI was used to determine whether the tumor thrombus was associated with bland thrombus (non-tumor thrombus), and the diagnostic criteria were based on our previous studies [15]. All patients were routinely performed preoperative abdominal B-ultrasound and abdominal enhanced CT to determine whether there were liver metastasis and adrenal metastasis, and lung CT plain scan was performed to determine whether there was lung metastasis. For those with bone pain or central nervous system symptoms, bone scan or head MRI should be improved. For patients with acceptable economic conditions, PETCT can be improved to comprehensively evaluate systemic metastasis. The American Society of Anesthesiologists (ASA) score was used to evaluate the general condition before anesthesia.

Surgical method: for the IVC tumor thrombus: (a) simple tumor thrombus removal: after vascular occlusion, the IVC wall was cut to remove the tumor thrombus, then the IVC wall was sutured. (b) Inferior vena cava transection: the tumor thrombus did not invade the vascular wall, but there was an extended non-neoplastic bland thrombus at the tumor thrombus’s distal end. After removing the tumor thrombus, the inferior vena cava was transected to prevent the bland thrombus from falling off [16]. (c) Segmental resection of the inferior vena cava: tumor thrombus invaded the vessel wall extensively, and the inferior vena cava was segmentally resected. Imaging diagnostic methods of tumor thrombus invasion of the vascular wall have been introduced in previous studies [17]. For right RCC with GADVR tumor thrombus: (a) simple left renal vein tumor thrombus removal: tumor thrombus did not invade the left renal vein; 2 Segmental resection of the left renal vein: tumor thrombus invaded left renal vein and segmental resection of invaded left renal vein. For left RCC with GADVR tumor thrombus, the left renal vein branches involved by the tumor thrombus were resected segmentally. Local lymph node dissection was performed in patients with enlarged lymph nodes by preoperative imaging. Postoperative complications were performed using the Clavien–Dindo surgical complication classification method, among which grades ≥ three were defined as severe complications [18].

Postoperative follow-up included survival, tumor recurrence or metastasis, postoperative renal function, and adjuvant drugs. Patients were asked to conduct an outpatient review every 3–6 months within 2 years after the operation, every 6–12 months after 2 years, and once a year after 5 years.

### Statistical analysis

The normality of continuous variables was tested, and the data of normal distribution were expressed as mean ± standard deviation; two independent samples T-test was used to analyze. For non-normal distribution data, they were expressed as median (IQR), using Mann–Whitney U test analysis. Categorical data were expressed by frequency (percentage), using Chi-square test, continuity correction, or Fisher’s exact test was used if the Chi-square test was not met. Univariate and multivariate Cox regression was performed to evaluate the prognostic significance of each variable relative to PFS. Kaplan–Meier plot was performed to evaluate the effect of GVDAR on progression-free survival (PFS), overall survival (OS) and cancer-specific survival (CSS), and P < 0.05 indicated that the difference was statistically significant. All statistical analyses were performed using SPSS 18.0.

## Results

We retrospectively analyzed the clinical characteristics of 21 patients with GADVR tumor thrombus and 192 patients with non-GADVR tumor thrombus in our center. Compared with non-GADVR tumor thrombus, patients with GADVR tumor thrombus had a higher proportion of open surgery (76.2% vs. 52.1%, P = 0.035), a higher proportion of tumor thrombus adhering to the inferior vena cava (IVC) vessel wall (81% vs. 45.8%, P = 0.002), a higher proportion of segmental resection of the IVC vessel wall (61.9% vs. 15.6%, P < 0.001); higher preoperative serum creatinine value (110.0 μmol/L vs. 91.0 μmol/L, P = 0.015), a higher proportion of tumor thrombus combined with bland thrombus (non-tumor thrombus) (57.1% vs. 19.8%, P < 0.001). In terms of surgical complexity, patients with GADVR tumor thrombus had a longer median operation time (379 min vs. 308 min, P = 0.038) and more median surgical blood loss (1400 mL vs. 600 mL, P = 0.018), and more postoperative complications (52.4% vs. 30.7%, P = 0.045). In other aspects, such as gender, age, clinical symptoms, BMI, Mayo classification, ASA grade, postoperative serum creatinine, tumor side, tumor diameter, lymph node metastasis and distant metastasis, pathological findings of sarcomatoid feature, perirenal fat infiltration, pathological type, nuclear grade, and severe postoperative complication, there was no significant difference between the two groups (see Table 1).

**Table 1:** Comparison of clinicopathological features between GADVR tumor thrombus and non-GADVR tumor thrombus

	non-GADVR (n = 192)	GADVR (n = 21)	P
Gender, n (%)			0.988
Male	146 (76.0%)	16 (76.2%)	
Female	46 (24.0%)	5 (23.8%)	
Age, y, median (IQR)	60.00 (14.00)	59.00 (10.00)	0.550
Clinical symptoms, n (%)			0.068
No	50 (26.0%)	1 (4.8%)	
Local symptoms	94 (49.0%)	16 (76.2%)	
Systemic symptoms	12 (6.2%)	1 (4.8%)	
Both	36 (18.8%)	3 (14.2%)	
BMI, kg/m^2^, mean ± SD	23.87 ± 3.91	24.95 ± 2.66	0.220
Surgical approach, n (%)			0.035^*^
Laparoscope	92 (47.9%)	5 (23.8%)	
Open	100 (52.1%)	16 (76.2%)	
Mayo classification, n (%)			0.093
0	54 (28.1%)	2 (9.5%)	
1	34 (17.7%)	3 (14.3%)	
2	70 (36.5%)	12 (57.2%)	
3	19 (9.9%)	4 (19.0%)	
4	15 (7.8%)	0 (0%)	
IVC segmental resection, n (%)			< 0.001^*^
No	162 (84.4%)	8 (38.1%)	
Yes	30 (15.6%)	13 (61.9%)	
Preoperative serum creatinine, µmol/L, median (IQR)	91.00 (27.00)	110.00 (34.00)	0.015^*^
Serum creatinine 1 week after operation, µmol/L, median (IQR)	97.00 (33.00)	96.00 (47.00)	0.739
Side, n (%)			0.392
Left	73 (38.0%)	10 (47.6%)	
Right	119 (62.0%)	11 (52.4%)	
Tumor diameter, cm, mean ± SD	8.94 ± 3.15	8.42 ± 2.52	0.472
Clinical N stage, n (%)			0.123
cN0	79 (41.1%)	5 (23.8%)	
cN1	113 (58.9%)	16 (76.2%)	
Adrenal metastasis, n (%)			0.613
No	176 (91.7%)	18 (85.7%)	
Yes	16 (8.3%)	3 (14.3%)	
Distant metastasis, n (%)			0.328
No	136 (70.8%)	17 (81.0%)	
Yes	56 (29.2%)	4 (19.0%)	
Bland thrombus, n (%)			< 0.001^*^
No	154 (80.2%)	9 (42.9%)	
Yes	38 (19.8%)	12 (57.1%)	
Sarcomatoid feature, n (%)			1.000
No	167 (87.0%)	18 (85.7%)	
Yes	25 (13.0%)	3 (14.3%)	
Perirenal fat infiltration, n (%)			0.728
No	130 (67.7%)	15 (71.4%)	
Yes	62 (32.3%)	6 (28.6%)	
Pathology type, n (%)			1.000
Clear cell RCC	160 (83.3%)	17 (81.0%)	
Non‐clear cell RCC	32 (16.7%)	4 (19.0%)	
Nuclear grade, n (%)			0.528
1	3 (1.6%)	0 (0%)	
2	70 (36.4%)	8 (38.1%)	
3	76 (39.6%)	11 (52.4%)	
4	43 (22.4%)	2 (9.5%)	
Lymph node dissection, n (%)			0.830
No	105 (54.7%)	12 (57.1%)	
Yes	87 (45.3%)	9 (42.9%)	
ASA grade, n (%)			0.816
1	13 (6.8%)	2 (9.5%)	
2	156 (81.2%)	17 (81.0%)	
3	23 (12.0%)	2 (9.5%)	
Operative time, min, median (IQR)	308.00 (158.00)	379.00 (138.00)	0.038^*^
Surgical blood loss, mL, median (IQR)	600.00 (1550.00)	1400.00 (850.00)	0.018^*^
Postoperative complication, n (%)			0.045^*^
No	133 (69.3%)	10 (47.6%)	
Yes	59 (30.7%)	11 (52.4%)	
Severe postoperative complication, n (%)			0.912
No	180 (93.8%)	19 (90.5%)	
Yes	12 (6.2%)	2 (9.5%)	
Thrombus adhering to the IVC, n (%)			0.002^*^
No	104 (54.2%)	4 (19.0%)	
Yes	88 (45.8%)	17 (81.0%)	

*GADVR* growing against the direction of venous return, *IQR* interquartile range, *BMI* body mass index, *SD* standard deviation, *RCC* renal cell carcinoma, *ASA* American Society of Anesthesiologists, *IVC* inferior vena cava

^*^p<0.05

The progression-free survival (PFS) was used as the prognostic outcome of this study, which refers to the time from the operation time to the first disease progression (Local recurrence or new metastasis occurred after operation for patients without preoperative metastasis, while local recurrence occurred after operation, and metastasis foci expanded or new metastasis occurred for patients with preoperative metastasis) or any cause of death [19]. Univariate Cox regression analysis found that GADVR tumor thrombus, clinical symptoms, surgical approach, IVC segmental resection, postoperative serum creatinine, distant metastasis, tumor thrombus combined with bland thrombus, sarcomatoid feature, perirenal fat infiltration, pathological type, nuclear grade, lymph node dissection, operation time and surgical blood loss were the risk factors for PFS. Multivariate Cox regression analysis showed that GADVR tumor thrombus, symptoms, postoperative serum creatinine, distant metastasis, sarcomatoid feature, pathological type, lymph node dissection were independent risk factors for PFS (see Table 2).

**Table 2:** Univariate and multivariate Cox regression analysis of prognostic factors for renal cell carcinoma with venous tumor thrombus

	Univariate			Multivariate		
	HR	95% CI	P	HR	95% CI	P
Gender	1.036	0.640–1.667	0.885			
Age	0.987	0.968–1.006	0.182			
Clinical symptoms						
No			Reference			Reference
Local symptoms	2.366	1.218–4.584	0.011^*^	1.944	0.942–4.009	0.072
Systemic symptoms	3.719	1.540–8.895	0.004^*^	2.974	1.183–7.474	0.020^*^
Both	4.375	2.143–8.932	0.000^*^	3.792	1.779–8.084	0.001^*^
BMI	0.956	0.906–1.009	0.100			
Surgical approach						
Laparoscope			Reference			
Open	1.907	1.233–2.950	0.004^*^	–	–	0.760
Mayo classification						
0			Reference			
1	0.589	0.271–1.280	0.181			
2	1.202	0.709–2.039	0.494			
3	1.907	0.959–3.796	0.066			
4	1.394	0.641–3.030	0.402			
IVC segmental resection	1.855	1.153–2.983	0.011^*^	–	–	0.571
Preoperative serum creatinine	1.003	0.994–1.011	0.540			
Serum creatinine 1 week after operation	1.002	1.001–1.004	0.010^*^	1.003	1.001–1.005	0.003^*^
Side						
Left			Reference			
Right	0.706	0.465–1.070	0.101			
Tumor diameter	1.059		0.077			
Clinical N stage						
cN0			Reference			
cN1	1.521	0.980–2.359	0.061			
Adrenal metastasis	1.425	0.737–2.756	0.293			
Distant metastasis	2.571	1.690–3.912	< 0.001^*^	2.571	1.580–4.010	< 0.001^*^
Bland thrombus	1.751	1.120–2.738	0.014^*^	–	–	0.632
Sarcomatoid feature	2.777	1.651–4.669	< 0.001^*^	2.698	1.541–4.809	0.001^*^
Perirenal fat infiltration	1.545	1.011–2.363	0.045^*^	–	–	0.802
Pathology type						
Clear cell RCC			Reference			
Non‐clear cell RCC	2.630	1.627–4.254	< 0.001^*^	2.206	1.286–3.783	0.004^*^
Nuclear grade						
1–2			Reference			
3–4	1.953	1.236–3.085	0.004^*^	–	–	0.098
Lymph node dissection	2.249	1.474–3.431	< 0.001^*^	1.981	1.271–3.087	0.003^*^
ASA grade						
1			Reference			
2	0.657	0.302–1.431	0.290			
3	0.814	0.320–2.069	0.665			
Operative time	1.002	1.001–1.004	0.002^*^	–	–	0.179
Surgical blood loss	1.000	1.000–1.000	0.013^*^	–	–	0.532
Postoperative complication	1.509	0.990–2.298	0.055			
Severe postoperative complication	1.833	0.948–3.543	0.071			
Thrombus adhering to the IVC	1.297	0.855–1.968	0.221			
GADVR	2.125	1.124–4.019	0.020^*^	2.039	1.030–4.038	0.041^*^

*HR* Hazard ratios, *CI* confidence interval, *BMI* body mass index, *IVC* inferior vena cava, *RCC* renal cell carcinoma, *ASA* American Society of Anesthesiologists, *GADVR* growing against the direction of venous return

^*^p<0.05

**Fig. 2:**
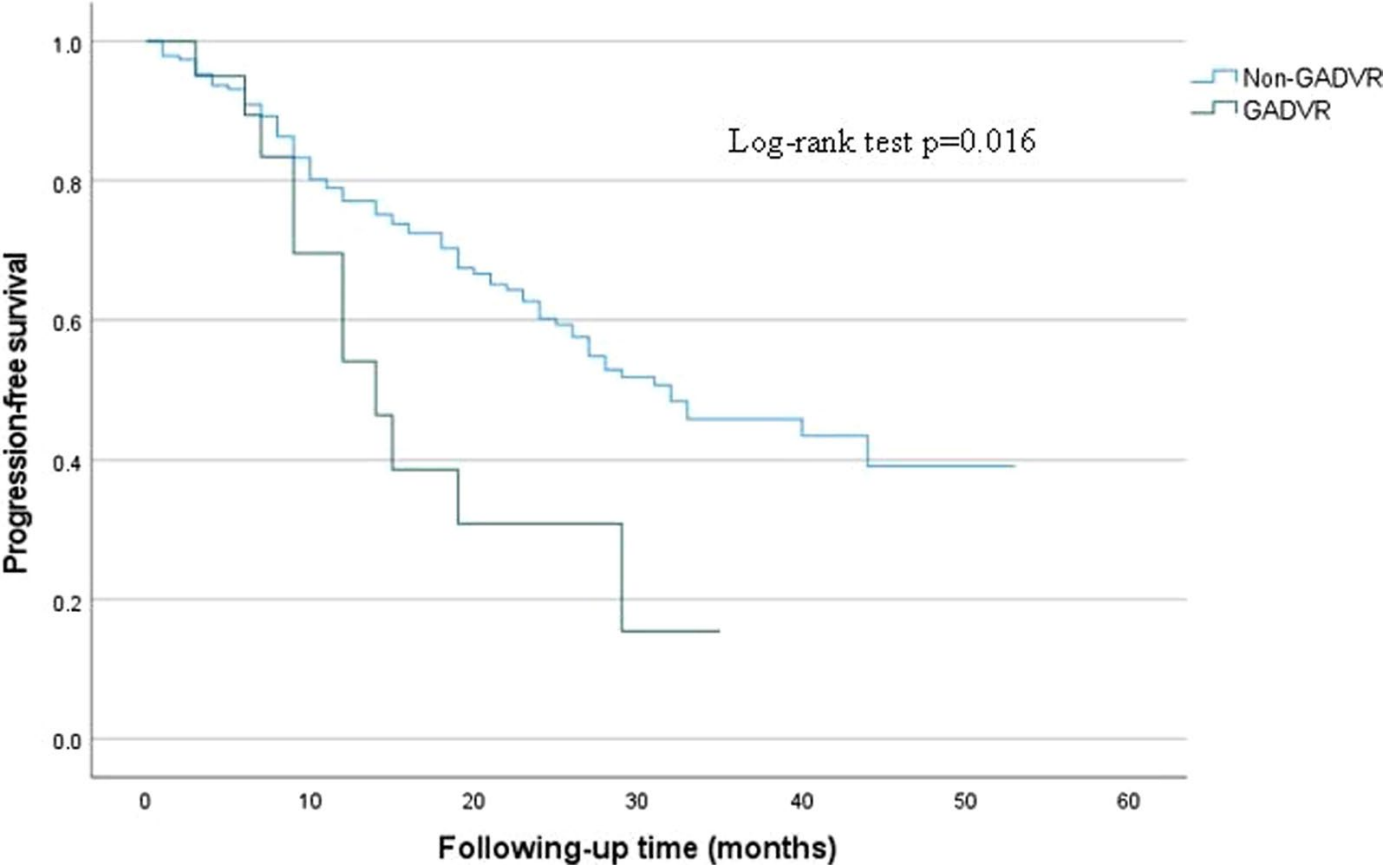
Progression-free survival of GADVR tumor thrombus and non-GADVR tumor thrombus. GADVR: growing against the direction of venous return

The survival curve analysis showed that the median PFS of patients with GADVR tumor thrombus was 14.0 months, while patients with non-GADVR tumor thrombus were 32.0 months (P = 0.016). However, there is no significant difference for OS (P = 0.958) and CSS (P = 0.794). During the follow-up period, only 4 of 21 GADVR patients died, of which 3 were tumor-specific deaths and 1 died of gastrointestinal bleeding. GADVR tumor thrombus is an independent risk factor affecting the PFS for RCC and VTT (see Fig. 2).

**Fig. 3:**
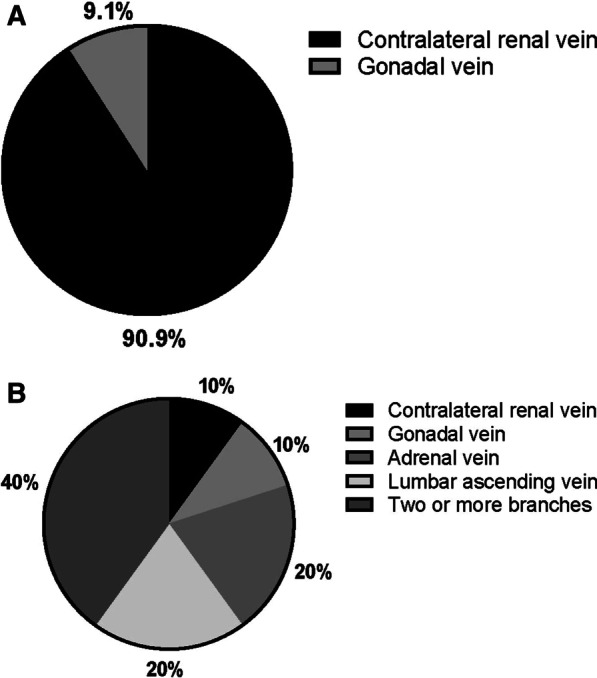
Types of affected veins in patients with GADVR tumor thrombus (pie chart). **A** Patients with right GADVR tumor thrombus; **B** Patients with left GADVR tumor thrombus. *GADVR* growing against the direction of venous return

Among patients with GADVR tumor thrombus, we divided them into right and left based on the primary renal tumor’s side, with 11 cases and 10 cases, respectively. Bilateral renal anatomy is different. The right renal vein usually has no branch, so the tumor thrombus usually grows to the left renal vein against the venous return. Most patients’ right gonadal vein flows directly into the inferior vena cava, and a few patients may mutate and flow into the right renal vein. Therefore, the GADVR tumor thrombus of right renal cancer may include the right gonadal vein tumor thrombus. The left renal vein usually has the left adrenal vein, the gonadal vein, the lumbar ascending vein as the branch, and the left GADVR tumor thrombus mainly manifests as the branch venous tumor thrombus. Comparison of clinicopathological features between right RCC with GADVR tumor thrombus and left RCC with GADVR tumor thrombus is shown in Table 3. We further classified GADVR tumor thrombus according to the different involved veins. Among the 11 patients with right GADVR tumor thrombus, ten patients (90.9%) extended to the left renal vein tumor thrombus, and one patient (9.1%) extended to the right gonadal vein. Among the ten patients with left GADVR tumor thrombus, there were one patient (10%) with right renal vein, one patient (10%) with left gonadal vein tumor thrombus, two patients (20%) with left adrenal vein tumor thrombus, two patients (20%) with lumbar ascending vein tumor thrombus, and four patients (40%) with two or more branches of tumor thrombus. In the above ten patients with left GADVR tumor thrombus, there were 15 venous tumor thrombus growing against the direction of venous return, including four (26.7%) in the left adrenal vein, five (33.3%) in the left gonadal vein, four (26.7%) in left lumbar ascending vein and two (13.3%) in the right renal vein (see Fig. 3).

**Table 3:** Comparison of clinicopathological features between right RCC with GADVR and left RCC with GADVR

	Right GADVR (n = 11)	Left GADVR (n = 10)	P
Gender, n (%)			0.635
Male	9 (81.8%)	7 (70.0%)	
Female	2 (18.2%)	3 (30.0%)	
Age, y, mean ± SD	55.09 ± 10.19	60.40 ± 6.69	0.179
Clinical symptoms, n (%)			1.000
No	0 (0%)	1 (10.0%)	
Local symptoms	8 (72.7%)	8 (80.0%)	
Systemic symptoms	1 (9.1%)	0 (0%)	
Both	2 (18.2%)	1 (10.0%)	
BMI, kg/m^2^, mean ± SD	25.18 ± 2.03	24.69 ± 3.32	0.687
Surgical approach, n (%)			0.635
Laparoscope	2 (18.2%)	3 (30.0%)	
Open	9 (81.8%)	7 (70.0%)	
Mayo classification, n (%)			0.430
0	0 (0%)	2 (20.0%)	
1	1 (9.1%)	3 (20.0%)	
2	7 (63.6%)	12 (50.0%)	
3	3 (27.3%)	4 (10.0%)	
IVC segmental resection, n (%)			0.080
No	2 (18.2%)	6 (60.0%)	
Yes	9 (81.8%)	4 (40.0%)	
Preoperative serum creatinine, µmol/L, mean ± SD	109.82 ± 20.89	99.40 ± 17.50	0.233
Serum creatinine one week after operation, µmol/L, median (IQR)	96.00 (45.00)	97.50 (49.00)	0.654
Tumor diameter, cm, mean ± SD	8.70 ± 2.77	8.12 ± 2.32	0.611
Clinical N stage, n (%)			0.311
cN0	4 (36.4%)	1 (10.0%)	
cN1	7 (63.6%)	9 (90.0%)	
Adrenal metastasis, n (%)			0.586
No	10 (90.9%)	8 (80.0%)	
Yes	1 (9.1%)	2 (20.0%)	
Distant metastasis, n (%)			0.035^*^
No	11 (100%)	6 (60.0%)	
Yes	0 (0%)	4 (40.0%)	
Bland thrombus, n (%)			0.030^*^
No	2 (18.2%)	7 (70.0%)	
Yes	9 (81.8%)	3 (30.0%)	
Sarcomatoid feature, n (%)			1.000
No	9 (81.8%)	9 (90.0%)	
Yes	2 (18.2%)	1 (10.0%)	
Perirenal fat infiltration, n (%)			1.000
No	8 (72.7%)	7 (70.0%)	
Yes	3 (27.3%)	3 (30.0%)	
Pathology type, n (%)			0.311
Clear cell RCC	10 (90.9%)	7 (70.0%)	
Non‐clear cell RCC	1 (9.1%)	3 (30.0%)	
Nuclear grade, n (%)			1.000
2	4 (36.4%)	4 (40.0%)	
3	6 (54.5%)	5 (50.0%)	
4	1 (9.1%)	1 (10.0%)	
Lymph node dissection, n (%)			1.000
No	6 (54.5%)	6 (60.0%)	
Yes	5 (45.5%)	4 (40.0%)	
ASA grade, n (%)			0.724
1	1 (9.1%)	1 (10.0%)	
2	8 (72.7%)	9 (90.0%)	
3	2 (18.2%)	0 (0%)	
Operative time, min, mean ± SD	338.18 ± 54.97	404.30 ± 125.70	0.150
Surgical blood loss, mL, median (IQR)	1500.00 (1100.00)	1000.00 (650.00)	0.085
Postoperative complication, n (%)			0.395
No	4 (36.4%)	6 (60.0%)	
Yes	7 (63.6%)	4 (40.0%)	
Severe postoperative complication, n (%)			1.000
No	10 (90.9%)	9 (90.0%)	
Yes	1 (9.1%)	1 (10.0%)	
Thrombus adhering to the IVC, n (%)			0.311
No	1 (9.1%)	3 (30.0%)	
Yes	10 (90.9%)	7 (70.0%)	

*GADVR* growing against the direction of venous return, *BMI* body mass index, *SD* standard deviation, *IQR* interquartile range, *RCC* renal cell carcinoma, *ASA* American Society of Anesthesiologists, *IVC* inferior vena cava

^*^p<0.05

## Discussion

In the clinical practice, we found a particular type of patients whose tumor thrombus is growing against the direction of venous return. We define it as GADVR tumor thrombus (Tumor thrombus growing against the direction of venous return). Veins are blood vessels that carry blood back to the heart. The ventricles draw blood from the atria and large veins during diastole. When inhaling, the pleural cavity’s negative pressure increases, and the pressure in the large veins in the thoracic cavity decreases, thereby promoting the return of venous blood [20]. Through the role of heart and respiration, for the left kidney, blood flows from the branch of the left renal vein to the left renal vein and then enters the IVC. For the right kidney, blood flows from the right renal vein into the IVC. GADVR tumor thrombus is a characteristic feature of tumor thrombus, a secondary manifestation of rapid tumor growth or high malignancy. The malignant degree of tumors in these patients is often high, and it is easy to cause tumor thrombus to obstruct the IVC. The space in the inferior vena cava is limited, limiting the proliferation and growth of tumors. Therefore, the tumor thrombus extends to the branch vein or contralateral renal vein, showing an abnormal growth pattern against the direction of venous return. For the right RCC and VTT, the GADVR tumor thrombus is manifested as the left renal vein tumor thrombus. For left RCC and VTT, the GADVR tumor thrombus showed left adrenal vein, left gonadal vein (testicular or ovarian vein), and left lumbar ascending vein tumor thrombus. Although GADVR tumor thrombus is a secondary manifestation, the importance of this concept has not been emphasized or reflected in previous studies. We came up with this concept because we found that patients with this type of surgery are more complicated and have a worse prognosis in clinical practice.

In this study, we found that GADVR tumor thrombus incidence was 9.9% in all patients with RCC and VTT. In terms of surgical approach, 76.2% of patients with GADVR tumor thrombus chose open surgery, while the proportion of patients with non-GADVR tumor thrombus was 52.1%. With the progress of minimally invasive technology, more and more centers have applied laparoscopic or robotic surgery, and the open approach is still a traditional and effective treatment. Our previous studies have found that the open approach is usually associated with large tumor load, severe adhesion, tumor thrombus invasion of the vascular wall, and full-filled tumor thrombus, which is a manifestation of complex surgery [21].

This study found that GADVR tumor thrombus had a higher proportion of adhesion to the IVC and IVC segmental resection. In clinical practice, we found that GADVR tumor thrombus is easy to obstruct the IVC. After the tumor thrombus adheres to the IVC, the inferior vena cava space is limited, limiting tumor proliferation and growth. Therefore, the tumor thrombus extends to the branch vein or contralateral renal vein, showing an abnormal growth pattern against the direction of venous return. In previous studies, we found that tumor thrombus combined with bland thrombus was a risk factor for surgical complexity and poor prognosis [15], and bland thrombus was also a manifestation of obstructive tumor thrombus [22]. We believe that the obstruction of tumor thrombus caused the slow blood flow, and the platelets and red blood cells in the blood gathered at the distal end of the tumor thrombus, resulting in long-term thrombosis; on the other hand, the obstruction caused the space limitation and formed the tumor thrombus against the direction of venous return. Therefore, it is considered that either tumor thrombus with bland thrombus or GADVR tumor thrombus formation is a secondary manifestation of tumor thrombus obstruction.

GADVR tumor thrombus has more operation time and more surgical blood loss in terms of surgical complexity, which requires clinicians to pay more attention. Before surgery, patients and their families should be fully communicated to inform them of surgical risks and get an understanding of patients. Although the incidence of severe complications was not significantly different from that of non-GADVR tumor thrombus, the overall incidence of postoperative complications was high, and more intensive care was needed after the operation.

In terms of prognosis, GADVR tumor thrombus is an independent risk factor affecting PFS. In this study, it was found that the median survival time of patients with GADVR tumor thrombus was 14.0 months, while that of patients with non-GADVR tumor thrombus was 32.0 months, and the difference was statistically significant. Patients with GADVR tumor thrombus should be followed up more closely after the operation. However, there is no significant difference for OS and CSS. Maybe because of the limited follow-up time and the limited number of dead patients, there was no significant difference in OS and CCS between GADVR patients and non-GADVR patients. Further expansion of sample size and longer follow-up are needed to improve the study in the future.

We distinguished 21 patients with GADVR tumor thrombus according to the type of vein involved. Before surgery, we confirmed GADVR tumor thrombus’s presence by urinary system enhanced CT or inferior vena cava enhanced MRI. Imaging findings usually show thickening of the branch vein with filling defect inside, and enhancement can be seen after the enhanced scan, which can be diagnosed as a branch tumor thrombus. Preoperative imaging is essential to determine the length of branch tumor thrombus to ensure sufficient resection of the involved vein. The operation will be as radical as possible to remove all tumor thrombus branches, along with the branch vein and its internal tumor thrombus, to ensure that the tumor resection clean, reduce the local recurrence rate. If necessary, a frozen pathological examination can be performed on the vein’s stump to ensure that the vascular wall margin is negative.

We acknowledge that there are some limitations in this study. Although the data comes from one of the largest RCC and VTT sample center in China, the incidence of GADVR in patients with RCC and VTT is about 9.9%, the number of GADVR patients included in this study is still limited. There may be uncontrollable confounding factors and inherent selection bias. In addition, this study is a single-center retrospective study with limited follow-up time, and future multi-center prospective studies need to be further designed for deeper pathological and genetic analysis.

## Conclusions

In summary, GADVR tumor thrombus is a characteristic feature of tumor thrombus, and its incidence is 9.9%. The proportion of open surgical approach is relatively high and the operation is more complex, which is an independent risk factor affecting PFS.

## Data Availability

The datasets used and/or analyzed during the current study are available from the corresponding author on reasonable request.

## Abbreviations

GADVR: Growing against the direction of venous return
RCC: Renal cell carcinoma
VTT: Venous tumor thrombus
PFS: Progression-free survival
OS: Overall survival
CSS: Cancer-specific survival
IVC: Inferior vena cava
ASA: American Society of Anesthesiologists
HR: Hazard ratios
CI: Confidence interval
SD: Standard deviation
IQR: Interquartile range
BMI: Body mass index
